# SARS-CoV-2 Structural Proteins Modulated Blood-Testis Barrier-Related Proteins through Autophagy in the Primary Sertoli Cells

**DOI:** 10.3390/v15061272

**Published:** 2023-05-29

**Authors:** Kai Kang, Yao-Dan Ma, Si-Qi Liu, Ri-Wei Huang, Jin-Jun Chen, Li-Long An, Jiang Wu

**Affiliations:** 1 Department of Veterinary Medicine, College of Coastal Agricultural Sciences, Guangdong Ocean University, Zhanjiang 524088, China; kangkai610@126.com (K.K.);; 2 Department of Animal Science, College of Coastal Agricultural Sciences, Guangdong Ocean University, Zhanjiang 524088, China

**Keywords:** SARS-CoV-2, blood-testis barrier, autophagy, Sertoli cells, junction protein, immune factor

## Abstract

The severe acute respiratory syndrome coronavirus-2 (SARS-CoV-2) disrupts the blood-testis barrier (BTB), resulting in alterations in spermatogenesis. However, whether BTB-related proteins (such as ZO-1, claudin11, N-cadherin, and CX43) are targeted by SARS-CoV-2 remains to be clarified. BTB is a physical barrier between the blood vessels and the seminiferous tubules of the animal testis, and it is one of the tightest blood-tissue barriers in the mammalian body. In this study, we investigated the effects of viral proteins, via ectopic expression of individual viral proteins, on BTB-related proteins, the secretion of immune factors, and the formation and degradation of autophagosomes in human primary Sertoli cells. Our study demonstrated that ectopic expression of viral E (envelope protein) and M (membrane protein) induced the expressions of ZO-1 and claudin11, promoted the formation of autophagosomes, and inhibited autophagy flux. S (spike protein) reduced the expression of ZO-1, N-cadherin, and CX43, induced the expression of claudin11, and inhibited the formation and degradation of autophagosomes. N (nucleocapsid protein) reduced the expression of ZO-1, claudin11, and N-cadherin. All the structural proteins (SPs) E, M, N, and S increased the expression of the FasL gene, and the E protein promoted the expression and secretion of FasL and TGF-β proteins and the expression of IL-1. Blockage of autophagy by specific inhibitors resulted in the suppression of BTB-related proteins by the SPs. Our results indicated that SARS-CoV-2 SPs (E, M, and S) regulate BTB-related proteins through autophagy.

## 1. Introduction

The coronavirus disease 2019 (COVID-19), caused by the severe acute respiratory syndrome coronavirus-2 (SARS-CoV-2), becomes a pandemic worldwide. The main symptoms of COVID-19 include various degrees of respiratory symptoms ranging from mild cough to chest pain and dyspnea [1]. Other symptoms include diarrhea [2], myocarditis [3], and neurological manifestations [4]. In addition, the SARS CoV-2 receptor angiotensin-converting enzyme 2 (ACE2) is highly expressed in the testicular tissue [5,6]. Although the SARS-CoV-2 viral particle was not detectable in semen [7,8], the viral RNA was detected in semen and testicular tissues [9]. Clinical reports showed that a moderate COVID-19 infection, without hospitalization, is associated with azoospermia for four weeks [10]. Autopsy reports indicated that a severe COVID-19 infection results in significant changes in the spatial arrangement of testicular cells, a reduction of BTB-related proteins, and an up-regulation of immune factors [11]. The genome of SARS-CoV-2 is a single-stranded, positive-sense RNA molecule that encodes 29 proteins, including four SPs (E, M, N, and S), 16 nonstructural proteins (Nsp1-16), and nine accessory proteins (ORFs). M, the envelope protein, determines viral shape. E interacts with M to form the viral membrane [12]. N, which forms the nucleocapsid, is closely related to testicular hormonal imbalance [13]. S, which binds to host cell receptors and mediates viral and host membrane attachment and fusion, was detectable in the endothelia of the BTB, spermatogenic cells, and stromal cells in the seminiferous tubules, and sperms in the epididymis [14]. Thus, the ability of SARS CoV-2 and its SPs to alter the male reproductive system needs to be further addressed.

Spermatogenesis occurs in a particular microenvironment composed of BTB and immune barriers. The BTB is a physical barrier between the blood vessels and the seminiferous tubules of the testis. The primary cells that form BTB are Sertoli cells, also known as ‘nurse’ cells, which provide nutrients, paracrine factors, cytokines, and other biomolecules to support germ cell development [15]. The adjacent Sertoli cells form the BTB through tight junctions (TJ), gap junctions (GJ), and adhesion junctions (AJ) [16]. The TJ, which is the main component of BTB, comprises occludin, ZO-1, and claudin [16]. The GJ-associated connexin-43 (CX43) maintains BTB integrity by regulating the expression and distribution of TJ-associated proteins [17]. Aberrant expression of the AJ-associated N-cadherin and β-catenin indicates BTB disruption [18,19]. The immune barrier-associated cytokines FasL, TGF-β, IL1α, and IL6, expressed in the Sertoli cells, play roles in modulating immune responses in the testis [20]. Both barriers of the physical BTB and the immune cytokines are essential for the homeostasis of spermatogenesis.

Autophagy plays a vital role in regulating cell growth, differentiation, and pathogenesis. Autophagy also participates in regulating BTB function. Induction of autophagy with zearalenone (ZEA) results in BTB destruction, and inhibition of autophagy with 3-methyladenine (3MA) or chloroquine (CQ) reduces the effects of ZEA on BTB [21]. SARS-CoV-2 causes autophagy to support optimal virus replication, and ectopic expression of the E protein activates autophagy, resulting in metabolic alterations, including the shutoff of protein synthesis and mobilization of cellular resources [22]. The ORF7a protein initiates autophagy and promotes viral replication [23], and inhibition of autophagy suppresses SARS-CoV-2 replication [24]. Accordingly, SARS-CoV-2 infection may induce autophagy to disrupt male reproductive ability [25]; however, the mechanism for SARS-CoV-2-induced autophagy in the alteration of BTB should be determined.

This research explored the effect of SARS-CoV-2 SPs on BTB-related proteins and autophagy in primary Sertoli cells. It also investigated the mechanism by which SARS-CoV-2-induced autophagy impairs BTB and the male reproductive system.

## 2. Materials and Methods

### 2.1. Cells and Plasmids

The primary human Sertoli cells (Sciencell, San Diego, CA, USA, Cat. #4520) were maintained in Sertoli Cell Medium (SerCM) with 5% heat-inactivated fetal bovine serum, 1% Sertoli Cell Growth Supplement, 100 U/mL penicillin, and 100 mg/mL streptomycin (Sciencell, San Diego, CA, USA) under 5% CO_2_ at 37 °C. The eukaryotic overexpression vectors of pEGFP-N1-HnCoV-E (GFP-E), pEGFP-N1-HnCoV-M (GFP-M), pEGFP-N1-HnCoV-N (GFP-N), and pEGFP-N1-HnCoV-S (GFP-S) were constructed by Shanghai General Biotech Co., Ltd. (Shanghai, China). The sequence information for the inserted DNA fragment is listed in Supplementary Materials.

### 2.2. Reagents and Antibodies

Rabbit antibodies specific to LC3 (L7543), ZO-1 (SAB5700645), occludin (SAB5700784), claudin 11 (ABT148), and pSQSTM1/P62 (SAB5700845) were purchased from Sigma-Aldrich (St. Louis, MO, USA). Rabbit antibodies specific to CX43 (A00599), N-cadherin (BA0673), and β-catenin (BA0426) were purchased from Boster (Wuhan, China). In addition, 3-methyladenine (3-MA) (A8780) was purchased from Solarbio (Beijing, China).

### 2.3. Cell Transfection

The Sertoli cells were seeded on 6-well plates. When 60–70% confluence was achieved, the cells were transfected with 2.5 μg plasmids [GFP-E, GFP-M, GFP-N, GFP-S, and pEGFP-N1 (GFP-V)] using 7.5 μL of Lipofectamine 3000 (Invitrogen, Carlsbad, CA, USA), respectively, according to the manufacturer’s instructions. At 48 h post-transfection, the cells were collected for transmission electron microscopy (TEM), immunoblotting, enzyme-linked immunosorbent assay (ELISA), quantitative real-time PCR (qPCR), and the cell supernatant was harvested for ELISA.

### 2.4. Biochemical Intervention

For autophagy inhibition experiments, Sertoli cells were seeded on 6-well plates and pretreated with 3 MA (5 mmol/mL) for 6 h. The inoculum was removed and washed twice with 0.01 M phosphate-buffered saline (PBS; pH 7.4), then transfected with 2.5 μg of plasmids (GFP-E, GFP-M, GFP-N, GFP-S, and GFP-V). The cells were then incubated in a fresh medium containing 3-MA for 48 h and subsequently collected for immunoblotting.

### 2.5. Transmission Electron Microscopy

Transfected Sertoli cells were digested by pancreatic enzymes, washed twice with PBS, centrifuged at 1500 r/min for 5 min, and the deposits were fixed by ice-cold glutaraldehyde at 4 °C for 1 h. The samples were dehydrated and embedded, then ultrathin sectioning images were observed and taken under the JEM-1400 transmission electron microscope (JEOL Ltd., Tokyo, Japan).

### 2.6. Reverse Transcription PCR (RT-PCR)

The total RNA of cell samples was extracted from Sertoli cells using Trizol reagent according to the manufacturer’s instructions (Solarbio, Beijing, China). First-strand cDNA was synthesized using a PrimeScript^TM^ RT reagent kit with gDNA Eraser (TaKaRa, Beijing, China). Primer GFP (Table 1) was designed according to the vector’s multiple cloning site to amplify the inserted genes of SARS-CoV-2 SPs in the pEGFP-N1 vector. The PCR reactions were performed in a total volume of 20 μL, containing 10 μL of 2× Es Taq MasterMix, 2 μL of cDNA template, 1 μL of forward and reverse primers, respectively, and 6 μL of sterile H_2_O. The reaction conditions were as follows: 94 °C for 5 min; 38 cycles at 94 °C for 30 s; 53 °C for 30 s; 72 °C for 4 min; and a final extension at 72 °C for 5 min. The results were analyzed using 1% agarose gel electrophoresis.

### 2.7. Quantitative Real-Time PCR (qPCR)

To examine the influence of SARS-CoV-2 SPs on genes’ expression, total RNA was prepared as in RT-PCR. cDNA was synthesized with 2 μg of total RNA using TransScript One-Step gDNA Removal and cDNA Synthesis SuperMix according to the reagent’s manual (TransGen Biotech, Beijing, China). qPCR was carried out using TB Green Premix Ex Taq II (Takara, Beijing, China) on a qPCR system (Bio-Rad, CA 94547, Hercules, CA, USA) with the following cycling profile: 5 min at 95 °C, followed by 40 cycles of 10 s at 95 °C, 20 s at 56 °C, and 20 s at 72 °C. The data were expressed as a relative fold change compared to the average value of the control group (GFP-V). The specific primer sequences (forward and reverse, respectively) for the reference genes were listed in Table 1. β-actin was used as an endogenous control gene.

### 2.8. Immunoblotting Analysis

To examine the influence of SARS-CoV-2 SPs on protein expression, transfected cells were digested by trypsin and suspended in 1 mL of PBS. Following centrifugation, cell pellets were resuspended in a cell lysis buffer (P0013, Beyotime, Shanghai, China) and centrifuged at 15,000× *g* for 10 min at 4 °C. The protein quantification of cell extracts was determined by the BCA Protein Assay Kit (P0009, Beyotime, Shanghai, China). The samples were separated by 12% sodium dodecyl sulfate polyacrylamide gel electrophoresis (SDS-PAGE). The proteins were transferred to a PVDF membrane (Millipore, Boston, MA, USA). The membrane was blocked with 5% skim milk in TBST for 1 h and then incubated with the first antibody at 4 °C overnight, then with the second antibody for 2 h. The protein bands were visualized with Clarity Western ECL Substrate (Bio-Rad, Hercules, CA, USA).

### 2.9. Enzyme-Linked Immunosorbent Assay (ELISA)

The Fas L, TGF β1, IL-1, and IL-6 in the transfected cells and supernatant were analyzed using commercial ELISA kits for humans (catalog numbers: Fas L, ml000971; TGF β1, ml013583; IL-1, ml001554; IL-6, ml001532; Shanghai Enzyme-linked Biotechnology Co., Ltd., Shanghai, China) following the manufacturer’s instructions.

### 2.10. Statistical Analysis

For TEM pictures, five cells from each sample were randomly selected for autophagosome structure counting. Data from relative qPCR were collected in triplicate and calculated using 2^−ΔΔCt^. Immunoblotting images and cell fluorescence photographs were the clearest ones from repeated performances and were quantified by NIH Image J software (ImageJ 149, NIH, Bethesda, MD, USA). Statistical differences between the means of the two groups were calculated using the Student’s *t*-test, and *p* values of <0.05 were considered to signify statistically significant differences. The means ± standard deviations were determined from at least three independent experiments.

## 3. Results

### 3.1. Transfection of SARS-CoV-2 Structural Proteins (SPs) in Primary Human Sertoli Cells

**Figure 1:**
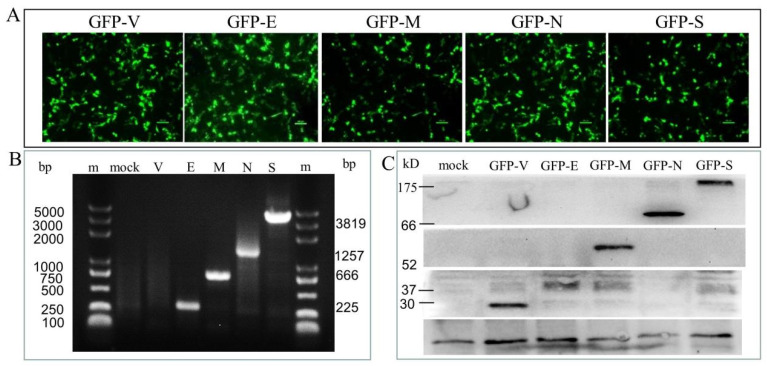
Identification of the expression of pGFP-SARS-CoV-2 SPs in the primary human Sertoli cells. (**A**) The expression of recombinant green fluorescent proteins (GFP-SPs) in Sertoli cells at 48 h post-transfection. (**B**) The nucleic acid expression levels of the SARS-CoV-2 NSPs, as analyzed by qPCR. Lane m is the DL5000 DNA marker; lane mock is the cells without transfection; lane V is the GPF vector control; lane E (225 bp), M (666 bp), N (1257 bp), and S (3819 bp). (**C**) The expression of the GFP-SARS-CoV-2 SPs, as analyzed by immunoblotting. Cell lysates were prepared from Sertoli cells expressing each of the SARS-CoV-2 E, M, N, and S and immunoblotted with antibodies against the GFP.

To study the function of each SARS-CoV-2 SP, we transiently transfected the primary human Sertoli cells with plasmid DNA to ectopically express GFP-tagged SPs (GFP-SPs), including GFP, GFP-E, GFP-M, GFP-N, and GFP-S. Ectopically expressed individual GFP-SPs in cells were detected under a fluorescence microscope (Figure 1A), gene expression of individual SPs was determined by the technique of RT-PCR (Figure 1B), and the protein level of ectopically expressed GFP-SPs was determined by immunoblotting (Figure 1C). These findings indicated successful transfection and expression of SARS-CoV-2 SPs in the primary Sertoli cells.

### 3.2. SARS-CoV-2 SPs Disrupt the Expression of BTB-Related Proteins

**Figure 2:**
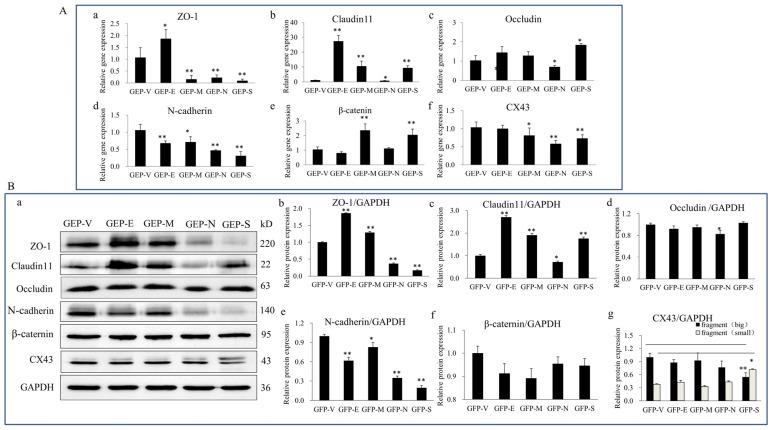
The effects of SARS-CoV-2 SPs on the expression of BTB-related proteins. (**A**) qPCR analysis of the mRNA expression of BTB-related genes. (**A-a**–**A-f**) Sertoli cells were transiently transfected with SARS-CoV-2 SP, then total RNAs were extracted for qPCR to analyze the mRNA expressions of ZO-1, claudin11, occludin, N-cadherin, β-catenin, and CX43. β-actin was the internal control. (**B**) Immunoblotting analysis of the expressions of BTB-related proteins. (**B-a**) Sertoli cells were transiently transfected with SARS-CoV-2 SPs, and then total cellular extracts were analyzed by immunoblotting of ZO-1, Claudin11, occludin, N-cadherin, β-Catenin, CX43, and GAPDH as the internal control. (**B-b**–**B-g**) The relative levels of the targeted proteins were shown by histograms representing density readings of the gel bands, and the ratios were calculated relative to the GAPDH control. The data represent the mean ± SD of three independent experiments. * *p* < 0.05, ** *p* < 0.01, calculated using the Student’s *t*-test of SARS-CoV-2 SPs transfected cells vs. empty plasmid (GFP-V) transfected cells.

To determine the effects of SARS-CoV-2 SPs on BTB-related proteins, Sertoli cells were transiently transfected with GFP-SARS-CoV-2 SPs plasmids for 48 h. qPCR analysis was used to detect the gene expression of TJ proteins ZO-1, occluding, and claudin-11; AJ proteins N-cadherin and β-catenin; and GJ proteins CX43. The results demonstrated that E induced gene expression of ZO-1 and claudin11 (Figure 2(A-a,A-b)) while reducing N-cadherin (Figure 2(A-d)). M induced gene expression of claudin11 and β-catenin (Figure 2(A-b,A-e)), while it reduced ZO-1, N-cadherin, and CX43 expression (Figure 2(A-a,A-d,A-f)). N reduced the gene expression of ZO-1, claudin11, occludin, N-cadherin, and CX43 (Figure 2(A-a–A-d)). S induced the gene expression of claudin11, occluding, and β-catenin (Figure 2(A-b,A-c,A-e)), while it reduced ZO-1, N-cadherin, and CX43 (Figure 2(A-a,A-d,A-f)).

We verified the level of protein expression by immunoblotting analysis to further substantiate these findings. The results showed that E and M proteins significantly enhanced the expression of ZO-1 and claudin11 (Figure 2(B-a–B-c)), while they reduced N-cadherin expression (Figure 2(B-a,B-e)). N and S significantly reduced the expression of ZO-1 and N-cadherin (Figure 2(B-a,B-b,B-e)). All the SPs reduced the expression of N-cadherin (Figure 2(B-a,B-e)). There were no significant changes in occludin, β-catenin, or CX43 expressions (Figure 2(B-a,B-d,B-f,B-g)). However, CX43 presented two closely migrating bands; the slower-migrating band was significantly enhanced in S ectopic expression cells (Figure 2(B-a,B-g)). To summarize, E and M enhanced the TJs proteins (ZO-1 and claudin11) expression while reducing the AJs proteins (N-cadherin). N and S significantly reduced the expression of TJ (ZO-1) and AJ (N-cadherin) proteins.

### 3.3. SARS-CoV-2 SPs Induce Expression of Immune Factors in Sertoli Cells

**Figure 3:**
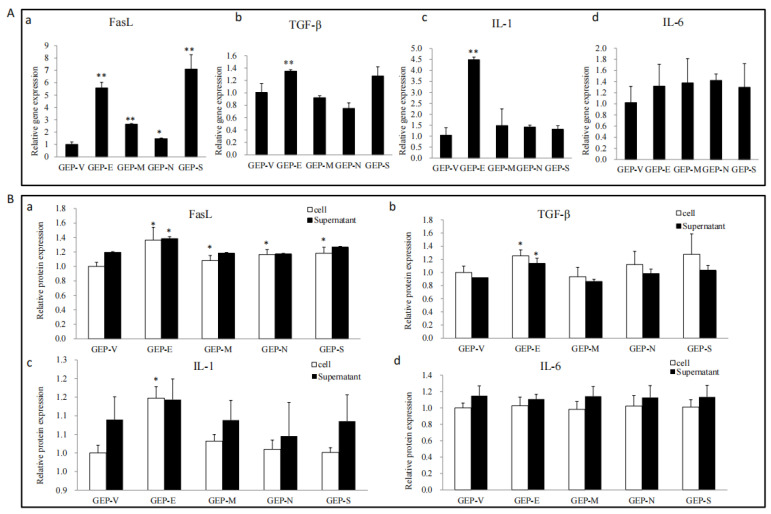
The effects of the SARS-CoV-2 SPs on the expressions of TGF-β, FasL, IL-1, and IL-6. (**A**) RT-PCR analysis of the mRNA expression of TGF-β, FasL, IL-1, and IL-6. Sertoli cells were transiently transfected with SARS-CoV-2 SPs for 48 h; then, total RNAs were extracted for qPCR to analyze the mRNA expression of the genes; β-actin was the internal control. (**B**) ELISA analysis of the expression and secretion of TGF-β, FasL, IL-1, and IL-6 proteins in Sertoli cells. Sertoli cells were transiently transfected with SARS-CoV-2 SPs for 48 h; the cells and culture supernatants were collected, respectively, and analyzed by ELISA. The results were presented in terms of relative expression in contrast to an empty plasmid (GFP-V). The data represent the mean ± SD of three independent experiments. * *p* < 0.05, ** *p* < 0.01, calculated using the Student’s *t*-test of SARS-CoV-2 SPs transfected cells vs. empty plasmid transfected cells.

As shown by the above results, SARS-CoV-2 SPs disrupted the physical barrier structure proteins of Sertoli cells, but whether SARS-CoV-2 SPs affected the immunomodulatory factors of Sertoli cells requires further studies. Here, TGF-β, FasL, IL-1, and IL-6, key molecules involved in Sertoli cell immunoregulation, were focused on [26]. The qPCR results showed that all the SPs induced FasL gene expression; in addition, E also induced the expressions of TGF-β, and IL-1 (Figure 3A).

To further determine protein expression and secretion, ELISA was used to test the expression of proteins in cell lysate and culture supernatant. The results showed that E enhanced the expression of FasL, TGF-β, and IL-1. All the SPs enhanced the expression of FasL and did not significantly affect the secretion of immune factors (Figure 3B).

### 3.4. SARS-CoV-2 SPs Influence on Sertoli Cells Autophagy

**Figure 4:**
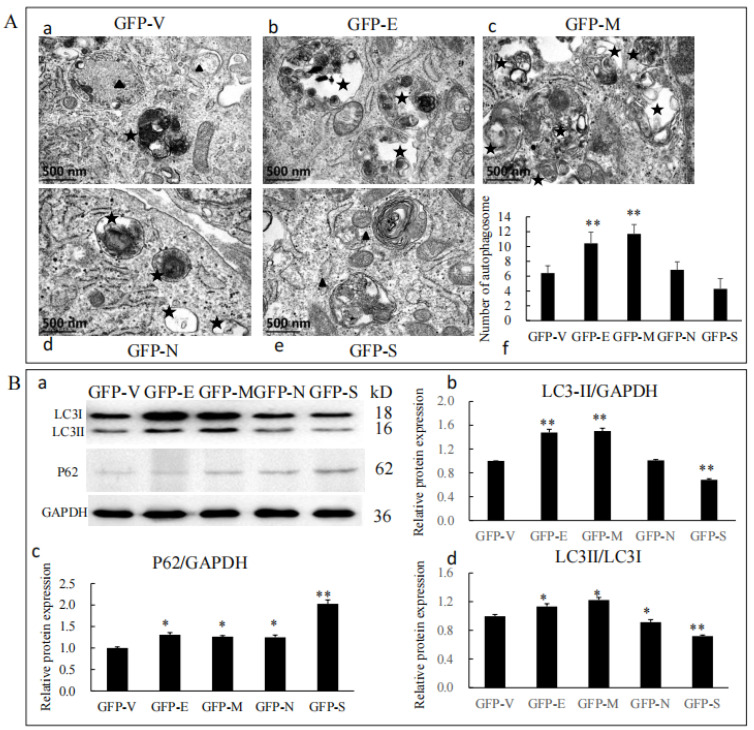
SARS-CoV-2 SPs influenced Sertoli cells’ autophagy. (**A**) TEM images of autophagic vacuoles in Sertoli cells; (**A-a**–**A-e**) cells were studied by electron microscopy at 48 h post-transfection with plasmids. The black triangles were autophagosomes for early autophagic vacuoles (AVi). The black stars were degradative autophagic vacuoles (AVd). (**A-f**) Quantification of the AVis and AVds per cell image. The average number of vesicles in each cell was obtained from at least five cells. (**B**) Immunoblotting analysis of the expressions of autophagy-related proteins. (**B-a**) The protein level of LC3 and P62 in Sertoli cells transiently transfected with SARS-CoV-2 structural proteins, with GAPDH serving as an internal control. (**B-b**–**B-d**) The relative levels of the targeted proteins were shown by histograms representing density readings of the gel bands, and the ratios were calculated relative to the GAPDH control. The data represent the mean ± SD of three independent experiments. * *p* < 0.05, ** *p* < 0.01, calculated using the Student’s *t*-test of SARS-CoV-2 SPs transfected cells vs. empty plasmid transfected cells.

To determine whether SARS-CoV-2 SPs modulate autophagy in Sertoli cells, we first examined the formation of autophagosome-like vesicles in SP-transfected cells using TEM and quantitative analyses (Figure 4A). There was a high background level of autophagy in primary Sertoli cells. It was easy to see the early autophagic vacuoles (AVi), with two bilayer vesicles and their contents of morphologically intact cytoplasm (black triangle), and the degradative autophagic vacuoles (AVd), with a high-density electron content (black stars) (Figure 4(A-a)). The number of AVi and AVd was significantly higher in E and M ectopic expressed cells, which were mainly AVd (Figure 4(A-b,A-c)). In N and S ectopic expressed cells, there was a similar number with an empty plasmid, which were mainly AVi (Figure 4((A-d,A-e)). Quantitative analysis also confirmed this (Figure 4(A-f)).

To further analyze whether SARS-CoV-2 SPs could trigger the autophagy machinery, we examined the expressions of P62, LC3, and LC3 conversion, which were widely used as markers for assessing the formation and degradation of autophagosomes [27,28]. The results showed that the protein level of LC3-II was increased by E and M but decreased by S (Figure 4(B-a,B-b)). LC3-II and LC3-I were increased by E and M but decreased by N and S (Figure 4(B-a,B-d)). All SARS-CoV-2 SPs increased the protein level of P62, and S showed significant differences (Figure 4(B-a,B-c)). Our results indicated that E and M induced the formation of autophagosomes but did not promote a complete autophagic flux. N and S proteins inhibited the formation of autophagosomes and impeded complete autophagy flux, resulting in the accumulation of p62 in cells.

### 3.5. Autophagy Inhibition Suppressed the Effects of SPs on BTB-Related Proteins

**Figure 5:**
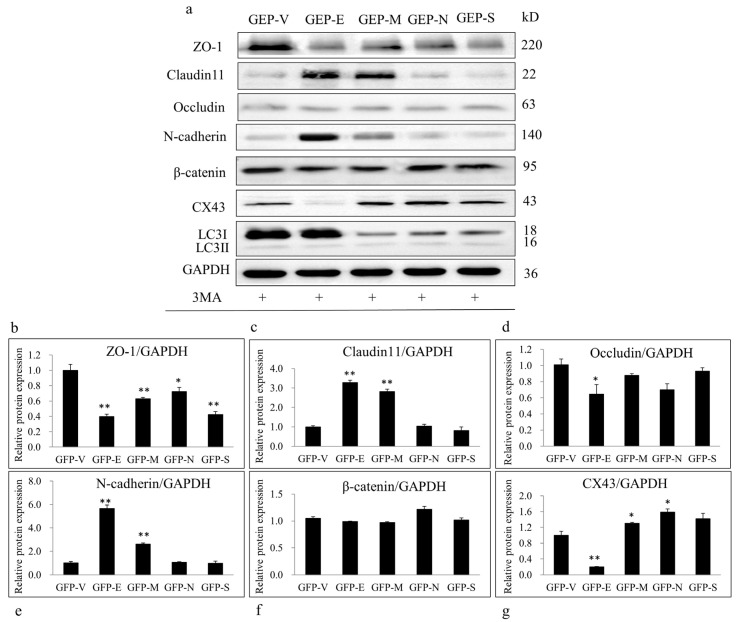
Inhibiting autophagy with 3-MA suppressed SARS-CoV-2 SPs’ effects on BTB-related proteins. (**a**) Sertoli cells were pretreated with 3-MA (5 mM) for 6 h, then transiently transfected with SARS-CoV-2 SPs. The expression of LC3, ZO-1, claudin11, occludin, N-cadherin, β-catenin, CX43, and GAPDH (internal control) was analyzed by immunoblotting with specific antibodies as described in Materials and Methods. (**b**–**g**) The relative levels of the targeted proteins were shown by histograms representing density readings of the gel bands, and the ratios were calculated relative to the GAPDH control. The data represent the mean ± SD of three independent experiments. * *p* < 0.05, ** *p* < 0.01, calculated using the Student’s *t*-test of SARS-CoV-2 SPs transfected cells vs. empty plasmid transfected cells.

To investigate whether autophagy was involved in SARS-CoV-2 SPs affecting BTB-related proteins, ST cells were treated with the autophagy inhibitor 3MA and then transfected with the SARS-CoV-2 SPs plasmids. The expressions of autophagy marker proteins and BTB-related proteins were examined by immunoblotting. After being treated with 3MA, the protein level of LC3-II significantly decreased in ST cells, indicating that autophagy was successfully inhibited (Figure 5a). Concerning the BTB-related proteins, the protein level of ZO-1 was significantly decreased by all SARS-CoV-2 SPs (Figure 5a,b), and the effects of E and M on ZO-1 expression were opposite to those of blank cells (Figure 2B). The protein level of claudin11 was significantly increased by E and M (Figure 5a,c), which was consistent with blank cells, and the use of 3MA inhibited the promotion effect of S protein on claudin11 expression (Figure 2B). The E protein reduced the expression of occludin (Figure 5a,d), which was opposite to that of blank cells (Figure 2B). E and M proteins significantly enhanced the expression of N-cadherin (Figure 5a,e), which was the opposite of blank cells (Figure 2B). All the proteins had the same effect on β-catenin as the cells without 3MA intervention (Figure 5a,f). E protein significantly reduced the expression of CX43, and M and N proteins enhanced the expression of CX43 (Figure 5a,g). These findings were the opposite of the blank cells. The electrophoretic variants of the S protein on CX43 were not visible (Figure 5a,g). All the results together led to the indicated conclusion that SARS-CoV-2 SPs modulate BTB-related proteins through autophagy.

## 4. Discussion

It is well known that SARS-CoV-2 uses ACE2 to invade human cells, and the high expression of AEC2 in Sertoli and germ cells makes the testis a potential target for infection [29]. It was also confirmed that SARS-CoV-2 disrupted the BTB and the expression of junctional proteins in vivo [11]. At present, the relationship between SARS-CoV-2 and BTB is still in the preliminary stage, and the effect of SARS-CoV-2 SPs on BTB at the molecular level is also unclear. This study investigated the effect of SARS-CoV-2 SPs on BTB-related proteins and the impact of autophagy on them.

The integrity of BTB is crucial to spermatogenesis because it is a physical barrier and provides an immune-privileged environment in vivo. Our findings showed that ectopic expression of SARS-CoV-2 SPs disrupted the expression of BTB-related proteins in Sertoli cells (Figure 2), indicating that SARS-CoV-2 posed a potential threat to BTB and could ultimately damage spermatogenesis. There were reports of viral infections destroying BTB, leading to semen poisoning. Mumps virus infecting Sertoli cells reduces occludin and ZO-1 levels, impairs BTB integrity, and disrupts BTB function, leading to male infertility [30]. ZIKV infection or E protein overexpression reduces the interaction between F-actin and ZO-1, disrupting the BTB and enhancing the permeability of the BTB [31]. The effects of SARS-CoV-2 on cellular junctional proteins are not limited to BTB. The reports of SARS-CoV-2 on the blood-gas barrier (BGB) showed that the virus triggered an inflammatory response, disassembly of AJs and TJs, and deposition of fibrin clots in alveolar epithelial cells (AECs) and endothelial cells (ECs), leading to the disintegration and thickening of the BGB [32]. Bioinformatics analyses of lung epithelial and alveolar cells with SARS-CoV-2 infection revealed 39 genes related to cell junctions, especially TJs [33]. The clinical sample demonstrated that SARS-CoV-2 could increase the blood-brain barrier (BBB) permeability and downregulate the TJs [34]. Further research showed that the S protein regulated the structure of the cell junction of the BBB, caused inflammatory responses, disrupted the function of the BBB, and caused neurological symptoms [35]. All the findings suggested that the modulation of cellular junctional proteins by SARS-CoV-2 was independent of cell and tissue type. In addition, we showed that, in addition to S, the N proteins also reduced the expression of junctional proteins. Bioinformatics analysis showed that the E protein had the structural basis for recognizing the cells’ junctional proteins [36], but E had no significant modulating function on junctional proteins in the present study. One interesting finding was that two closely migrating bands were seen on CX43, and the S protein significantly enhanced the expression of the large band while the small band was reduced (Figure 2). CX43 is a protein of the gap junction channel, formed by docking two hexametric hemichannels. An explanation was that the S protein affected CX43 assembly. CX43 did not have alternative splicing but had transcriptional factor activity to directly regulate the transcription of N-cadherin [37]. The protein in the nucleus was shown as two distinct bands [38], and the expression of N-cadherin was significantly reduced in S-transfected cells. Hence, we suggest another hypothesis that the S reduced the expression of CX43 in the membrane, enhanced CX43 phosphorylation and transfer into the nucleus, and interfered with the structure of the gap junction. Further investigations are required to shed light on this aspect.

In recent years, autophagy has been demonstrated to be involved in various physiological functions in vitro and disease responses in vivo, such as the replication of viruses, cell differentiation, and regulating the course of diseases. Our findings suggested that SARS-CoV-2 SPs affected the expression of BTB-related proteins through autophagy. The effects of E, M, and S on the expression of BTB-related proteins were altered when autophagy was inhibited (Figure 2 and Figure 5). Autophagy regulated the BTB’s structure and barrier function; di-(2-ethylhexyl) phthalate (DEHP) exposure destroyed rats’ BTB integrity, down-regulated junctional proteins, and induced the number of autophagosomes and the levels of autophagy markers LC3-II and p62. Inhibition of autophagy by CQ and 3-MA was sufficient to reduce the effects of DEHP on BTB [39]. Our findings showed that E and M induced autophagy formation (Figure 4A) but impeded autophagy flux (Figure 4B). Adding 3MA suppressed the effects of E and M on BTB-related proteins ZO-1, N-cadherin, and β-catenin, and S on cluadin11 and CX43 (Figure 5). Our findings indicated that the modulatory effects of E, M, and S on BTB are mediated by autophagy. The N reduced multiple BTB proteins’ expression, but the regulatory pathway was independent of autophagy. It was reported that the E and M proteins lead to the accumulation of autophagosomes, but the M protein did not alter P62 protein levels [40], which is different from our results. In a hamster model and lung samples of COVID-19 patients, phagophore-incorporated autophagy markers LC3-II and P62 accumulated, but the results indicated that the accumulation was caused by autophagy inhibition [41]. In another report, the M protein induced mitophagy by interacting with LC3 [42]. These findings may not seem entirely consistent, but there was one commonality: SARS-CoV-2 caused the accumulation of autophagosomes in vivo and in vitro. We believe that the regulation of autophagy by SARS-CoV-2 indicates differences in tissues and dependence on the cell type in vitro. Single-nucleus and single-cell sequencing of patient-derived lung and mucosal samples also confirmed this [41].

The immunomodulatory effect of Sertoli cells plays an essential role in maintaining the normal function of the BTB. Sertoli cells produce a variety of cytokines, including chemokines, growth factors, inflammatory mediators, complement inhibitors, and adhesion molecules, to regulate the immune response of the testes [20]. TGF-β, FasL, IL-1α, and IL-6 were confirmed to regulate testis immunity and maintain tissue immune privileges [20]. In a herbicide model, 2, 4-dichlorophenoxyacetic acid (2, 4-D) induced testicular injury and mouse Setoli cell (TM4) apoptosis, and the expression of Fas and FasL was significantly upregulated. Depletion of Fas by specific shRNA transfection reversed the effects of 2, 4-D in TM4 cells [43]. TGF-β modulates immune factors to inhibit the immune response in the testis [44]. In a model of orchitis induced by LPS in bovine Sertoli cells, LPS induced IL-6 and IL-1 β, and downregulated the expression of ZO-1 and occludin, resulting in the inflammatory response of Sertoli cells and TJ damage [45]. The immunomodulatory effect of Sertoli cells was also used in allotransplantation to prolong the transplantation time and improve the success rate [46]. All the evidence suggested that the ability of Sertoli cells to immunomodulate was critical for BTB. We tested the effects of SARS-CoV-2 SPs on the expression and secretion of TGF-β, FasL, IL-1, and IL-6. The results showed that all SARS-CoV-2 SPs could induce the expression of FasL (Figure 3), a pro-apoptotic molecule called Fas ligand that could induce cell apoptosis. FasL inhibits the testis immune response by inducing immune cells, such as lymphocytes, to undergo apoptosis in the testis. SARS-CoV-2 triggers the Fas/FasL signaling pathway to promote apoptosis as one of the important ways to cause pathological signs [47]. This was consistent with our results. E protein induced the expression of TGF-β and IL-1. The rest of the proteins had no significant effects on the expression and secretion of TGF-β, IL-1, or IL-6 (Figure 3). Multiple reports have shown that the SARS-CoV-2 infection caused a cytokine storm in the body and triggered an inflammatory response, even in adipose tissue [48]. A single-cell sequencing of alveolar epithelial cells showed that SARS-CoV-2 induced IL-6 expression [49], and IL-6 has widely been acknowledged to play an important role in COVID-19. However, our findings showed that SARS-CoV-2 SPs did not significantly increase IL-6 and IL-1 in Sertoli cells. It is indicated that SARS-CoV-2 causes different inflammatory responses in vivo and in vitro, or maybe other viral proteins have the function of modulating immune factors.

## 5. Conclusions

Ectopic expression of SARS-CoV-2 SPs (E, M, N, and S) in primary human Sertoli cells modulated the expression of BTB-related proteins and autophagy and increased the expression of FasL. Autophagy mediates the effect of SARS-CoV-2 SP on BTB-related proteins. The E and M proteins induced the expression of BTB-related proteins ZO-1 and Claudin11, promoted the formation of autophagosomes, and impeded autophagic flux. On the contrary, the S protein reduced the expression of BTB-related proteins ZO-1 and N-cadherin and inhibited autophagosome degradation. The suppression of autophagy with 3MA showed that the effect of E, M, and S proteins on the BTB-related proteins was mediated by autophagy. These findings make it necessary to study the effect of SARS-CoV-2 on BTB in vivo and extend research on the SARS-CoV-2’s effect on cell junctions in other cell types.

## Figures and Tables

**Table 1:** The sequence of primers used in this study.

Genes	Forward Primer (5′–3′)	Reverse Primer (5′–3′)
ZO-1	CGGTGGTAACTTTGAGA	TCTGAGATGGAGGTGGGT
Occludin	GTGCCATCATTGCGGGATTC	AGGTGGATATTCCCTGA
Claudin11	TGTTGGGCTTCATTCTCG	GGCGGTCACGATGTTGT
β-catenin	GGTCCGAGTGCTGCTCATG	GCTGTCAGGTTTGATCCCATC
N-cadherin	CTGAAGCCAACCTTAACTGA	TGTCCCATTCCAAACCTG
CX43	TCGCCTATGTCTCCTCCTG	AGGTCGCTGGTCCACAAT
FasL	GTTCTGGTTGCCTTGGTA	GTGGCCTATTTGCTTCTC
TGF-β1	TCCACGGAGAAGAACTGC	CAGGCTCCAAATGTAGGG
IL-1	AGTGCTGCTGAAGGAGAT	TGGATGGGCAACTGATGT
IL-6	GGAGACTTGCCTGGTGAA	AGCTCTGGCTTGTTCCTC
β-actin	GAAATCGTGCGTGACATCAAAG	TGTAGTTTCATGGATGCCACAG
GFP	CTCAGATCTCGAGCTCAAGC	TGGCGACCGGTGGATC

## Data Availability

The authors confirm that all the data used in the article supporting this study is available within the article.

## Supplementary Materials

The following supporting information can be downloaded at: https://www.mdpi.com/article/10.3390/v15061272/s1, title: Attachment-gene sequence (The material is also available online at https://zenodo.org/deposit/7937011).
