# Supplementary material for: SARS-CoV-2 Structural Proteins Modulated Blood-Testis Barrier-Related Proteins through Autophagy in the Primary Sertoli Cells
Source: Viruses. 2023 May 29;15(6):1272. doi: 10.3390/v15061272 (PMC10304867; doi:10.3390/v15061272)
Supplement: Supplementary file 1 [file viruses-15-01272-s001.zip › viruses-2429252-supplementary.pdf]

Plasmid: pEGFP-N1, Endonuclease enzyme: EcoRI/BamHI

SARS-CoV-2-E
(https://www.ncbi.nlm.nih.gov/nucleotide/OM273378.1?report=genbank&log$=nuclalign&blast_rank=1&RID=SAPP56TB013)

GAATTCGCCACCATGTACAGCTTCGTGAGCGAGGAGACCGGCACACTGATTGTGAACTCCGTGCTGCTCTTCCTGGCCT
TCGTGGTGTTCCTGCTCGTGACACTCGCCATTCTGACCGCCCTCAGACTGTGCGCCTACTGCTGCAACATTGTGAACGT
GTCCCTGGTGAAGCCATCTTTCTACGTGTACTCCAGAGTGAAGAACCTCAACAGCTCTAGGGTGCCCGACCTCCTGGTG
CGGGATCC

SARS-CoV-2-M:
(https://www.ncbi.nlm.nih.gov/protein/8CTK_A?report=genbank&log$=protalign&blast_rank=1&RID=SARPHOF1013)

GAATTCGCCACCATGGCCGACTCTAACGGCACAATTACAGTGGAGGAGCTGAAGAAGCTGTTAGAACAGTGGAACCTGG
TGATCGGCTTCCTGTTCCTGACATGGATTTGCCTCCTCCAGTTCGCCTACGCCAACCGGAACAGGTTCCTGTACATTAT
TAAGCTGATTTTCCTGTGGCTCCTCTGGCCCGTGACACTGGCCTGCTTCGTGCTCGCCGCCGTGTACCGGATTAACTGG
ATCACAGGCGGCATTGCCATTGCGATGGCCTGCCTCGTGGGCCTCATGTGGCTGTCTTACTTCATTGCCTCTTTCAGAC
TGTTCGCCCGCACACGGTCTATGTGGTCTTTCAACCCAGAGACAAACATTCTGCTGAACGTGCCCCTGCACGGCACCAT
TCTCACAAGACCACTGCTTGAATCCGAGCTGGTGATCGGCGCCGTGATTCTGAGAGGCCACCTCAGAATTGCCGGCCAC
CACCTGGGCAGATGCGACATCAAGGACCTCCCTAAGGAGATCACAGTGGCCACATCTAGGACACTCAGCTACTACAAGC
TGGGCGCCTCGCAGCGAGTGGCCGGCGACTCCGGCTTCGCCGCCTACAGCCGTTACCGCATCGGCAACTACAAGCTCAA
CACCGACCACTCTTCTAGCAGCGACAACATCGCCCTGCTCGTGCAGCGGGATCC

SARS-CoV-2-N
(https://www.ncbi.nlm.nih.gov/nucleotide/MZ948831.1?report=genbank&log$=nuclalign&blast_rank=1&RID=SAPZ4RBG013)

GAATTCGCCACCATGAGCGACAACGGCCCTCAGAACCAGCGGAACGCCCCTAGGATTACATTCGGCGGCCCATCTGACT
CTACCGGCTCTAACCAGAACGGCGAGCGATCCGGCGCCCGGTCCAAGCAGCGGAGACCTCAGGGCCTGCCAAACAACAC
AGCCTCTTGGTTCACAGCCCTCACCCAGCACGGCAAGGAGGACCTGAAGTTCCCTAGAGGCCAGGGCGTGCCAATTAAC
ACAAACTCTTCTCCTGACGACCAGATTGGCTACTACAGACGCGCCACACGGAGAATCCGGGGCGGCGACGGCAAGATGA
AGGACCTGTCTCCTAGGTGGTACTTCTACTACCTGGGCACCGGCCCCGAGGCCGGCCTCCCATACGGCGCCAACAAGGA
CGGCATTATTTGGGTGGCCACCGAGGGCGCCCTGAACACCCCAAAGGACCACATTGGCACACGGAACCCTGCCAACAAC
GCCGCCATCGTGCTACAGCTCCCTCAGGGCACCACACTGCCTAAGGGCTTCTACGCCGAGGGCTCTCGGGGCGGCTCTC
AGGCCTCTTCTAGGTCTAGCTCTCGTTCTCGCAACTCTTCCCGGAACTCCACCCCTGGCTCTTCCCGAGGCACATCTCC
TGCCCGCATGGCCGGCAACGGCGGCGACGCCGCCCTCGCCCTGCTCCTGCTGGACAGACTGAACCAGCTGGAGTCTAAG
ATGTCTGGCAAGGGCCAGCAGCAGCAGGGCCAGACCGTGACAAAGAAGTCCGCCGCCGAGGCCTCTAAGAAGCCTAGAC
AGAAGCGCACAGCCACAAAGGCCTACAACGTGACACAGGCCTTCGGGCGGAGAGGCCCCGAGCAGACACAGGGCAACTT
CGGCGACCAGGAGCTGATTAGACAGGGCACCGACTACAAGCACTGGCCTCAGATTGCCCAGTTCGCCCCTAGCGCCTCT
GCCTTCTTCGGCATGTCTCGCATTGGCATGGAGGTGACCCCCAGCGGGACGTGGCTCACATACACCGGCGCCATTAAGC
TGGACGACAAGGACCCAAACTTCAAGGACCAGGTGATTCTGCTGAACAAGCACATTGACGCCTACAAGACATTCCCACC
TACCGAGCCAAAGAAGGACAAGAAGAAGAAGGCCGACGAGACCCAGGCCCTCCCTCAGAGACAGAAGAAGCAGCAGACC
GTGACACTGCTCCCCGCCGCCGACCTCGACGACTTCTCTAAGCAGCTTCAGCAGTCCATGTCTTCTGCCGACTCCACCC
AGGCCCGGGATCC

SARS-CoV-2-S：
(https://www.ncbi.nlm.nih.gov/nucleotide/MZ393690.1?report=genbank&log$=nuclalign&blast_rank=1&RID=SAR32RX7016)

GAATTCGCCACCATGTTCGTGTTCCTCGTGCTGCTCCCACTGGTGTCTAGCCAGTGCGTGAACCTCACCACCCGCACCC
AGCTCCCTCCTGCCTACACAAACAGCTTCACAAGGGGCGTGTACTACCCTGACAAGGTGTTCCGGTCTAGCGTGCTGCA
CTCTACACAGGACCTGTTCCTCCCTTTCTTCTCTAACGTGACATGGTTCCACGCCATCCATGTATCCGGCACAAACGGC
ACAAAGCGGTTCGACAACCCCGTGCTCCCTTTCAACGACGGCGTGTACTTCGCCAGCACCGAGAAGTCTAACATTATTA
GAGGCTGGATTTTCGGCACCACACTCGACTCTAAGACCCAGTCTCTCCTGATTGTGAACAACGCCACAAACGTGGTGAT
TAAGGTGTGCGAGTTCCAGTTCTGCAACGACCCTTTCCTGGGCGTGTACTACCACAAGAACAACAAGTCTTGGATGGAG
TCCGAGTTCCGGGTGTACTCTAGCGCCAACAACTGCACATTCGAGTACGTGTCTCAGCCTTTCCTCATGGACCTGGAGG
GCAAGCAGGGCAACTTCAAGAACCTGAGAGAGTTCGTGTTCAAGAACATTGACGGCTACTTCAAGATTTACTCCAAGCA
CACCCCAATTAACCTGGTGAGGGACCTCCCTCAGGGCTTCTCCGCCCTGGAACCCCTCGTGGACCTCCCAATTGGCATT
AACATCACCCGGTTCCAGACACTGCTCGCCCTCCACCGGTCTTACCTGACCCCAGGCGACTCTTCTAGCGGCTGGACAG
CCGGCGCCGCCGCCTACTACGTGGGCTACCTCCAGCCACGCACATTCCTCCTGAAGTACAACGAGAACGGCACAATTAC
CGACGCCGTGGACTGCGCCCTCGACCCACTGAGCGAGACAAAGTGCACACTGAAGTCTTTCACAGTGGAGAAGGGCATT
TACCAGACATCTAACTTCCGGGTGCAGCCTACCGAGTCTATTGTGCGGTTCCCAAACATTACCAACCTGTGCCCTTTCG
GCGAGGTGTTCAACGCCACCCGCTTCGCCTCTGTGTACGCCTGGAACCGGAAGCGGATTTCTAACTGCGTGGCCGACTA
CTCTGTGCTGTACAACAGCGCCTCTTTCTCCACATTCAAGTGCTACGGCGTGTCCCCTACAAAGCTGAACGACCTGTGC
TTCACAAACGTGTACGCCGACTCTTTCGTGATCCGGGGCGACGAGGTGAGGCAGATTGCCCCAGGCCAGACAGGCAAGA
TTGCCGACTACAACTACAAGCTCCCTGACGACTTCACAGGCTGCGTGATCGCCTGGAACTCTAACAACCTCGACTCTAA
GGTGGGCGGCAACTACAACTACCTGTACAGACTGTTCCGCAAGTCTAACCTGAAGCCTTTCGAGCGGGACATTAGCACC
GAGATTTACCAGGCCGGCAGCACCCCATGCAACGGCGTGGAGGGCTTCAACTGCTACTTCCCACTCCAGTCTTACGGCT
TCCAGCCTACAAACGGCGTGGGCTACCAGCCATACCGGGTGGTGGTGCTGTCTTTTGAATTGCTCCACGCCCCCGCCAC
AGTGTGCGGCCCTAAGAAGTCCACAAACCTGGTGAAGAACAAGTGCGTGAACTTCAACTTCAACGGCCTCACAGGCACA
GGCGTGCTCACCGAGTCCAACAAGAAGTTCCTGCCTTTCCAGCAGTTTGGCCGGGACATTGCCGACACCACCGACGCCG
TGAGGGACCCTCAGACATTGGAGATTCTGGACATCACCCCATGCAGCTTCGGCGGCGTGTCTGTGATCACCCCCGGCAC
CAACACATCTAACCAGGTGGCCGTGCTGTACCAGGACGTGAACTGCACAGAGGTGCCAGTGGCCATCCACGCCGACCAG
CTGACCCCTACATGGAGGGTGTACTCTACAGGCTCTAACGTGTTCCAGACTCGCGCCGGCTGCCTGATTGGCGCCGAGC
ACGTAAACAACAGCTACGAGTGCGACATCCCAATTGGCGCCGGCATTTGCGCCTCTTACCAGACCCAGACAAACTCTCC
TAGACGGGCCCGGTCTGTGGCCTCTCAGTCTATTATTGCCTACACCATGTCCCTGGGCGCCGAGAACTCTGTGGCCTAC
TCTAACAACTCTATTGCCATCCCTACAAACTTCACAATTTCTGTGACCACCGAGATTCTCCCAGTGTCTATGACAAAGA
CATCCGTGGACTGCACCATGTACATTTGCGGCGACTCTACCGAGTGCTCTAACCTCCTGCTCCAGTACGGCTCTTTCTG
CACCCAGCTCAACCGGGCCCTCACAGGCATTGCCGTGGAGCAGGACAAGAACACACAGGAGGTGTTCGCCCAGGTGAAG
CAGATTTACAAGACCCCTCCTATTAAGGACTTCGGCGGCTTCAACTTCTCTCAGATTCTCCCCGACCCTAGCAAGCCAT
CTAAGCGGTCCTTCATTGAGGACCTGCTGTTCAACAAGGTGACACTGGCCGACGCCGGCTTCATTAAGCAGTACGGCGA
CTGCCTGGGCGACATCGCCGCTCGTGACCTGATTTGCGCCCAGAAGTTCAACGGCCTGACAGTGCTCCCCCCACTGCTC
ACCGACGAGATGATTGCCCAGTACACCAGCGCCCTCCTCGCCGGCACCATCACATCTGGCTGGACCTTCGGCGCCGGCG
CCGCCCTACAGATCCCTTTCGCCATGCAGATGGCCTACCGCTTCAACGGCATCGGCGTGACCCAGAACGTGCTGTACGA
GAACCAGAAGCTCATCGCCAACCAGTTCAACTCTGCCATTGGCAAGATTCAGGACTCTCTGTCTAGCACAGCCAGCGCC
CTGGGCAAACTGCAAGACGTGGTGAACCAGAACGCCCAGGCCCTCAACACACTGGTGAAGCAACTCTCGTCTAACTTCG
GCGCCATTAGCTCTGTGCTGAACGACATTCTGTCCCGACTCGACAAGGTGGAGGCCGAGGTGCAGATTGACAGACTGAT
TACAGGCAGACTCCAATCTCTACAGACATACGTGACACAGCAGCTCATTAGAGCCGCCGAGATCAGAGCCTCCGCCAAC
CTGGCCGCCACAAAGATGAGCGAGTGCGTGCTGGGCCAGTCCAAGAGAGTGGACTTCTGCGGCAAGGGCTACCACCTCA
TGTCTTTCCCTCAGTCCGCCCCTCACGGCGTGGTGTTCCTCCATGTTACATACGTGCCAGCCCAGGAGAAGAACTTCAC

CACAGCCCCCGCCATTTGCCACGACGGCAAGGCCCACTTCCCTAGAGAGGGCGTGTTCGTGTCCAACGGCACCCACTGG
TTCGTGACCCAGAGAAACTTCTACGAGCCTCAGATTATCACCACCGACAACACCTTCGTGTCTGGCAACTGCGACGTGG
TGATTGGCATTGTGAACAACACAGTGTACGACCCATTGCAACCCGAACTTGACTCTTTCAAGGAGGAACTTGACAAGTA
CTTCAAGAACCACACATCTCCCGACGTGGACCTGGGCGACATTAGCGGCATTAACGCCTCTGTGGTGAACATTCAGAAG
GAGATCGACAGGCTGAACGAGGTGGCCAAGAACCTGAACGAGTCTCTGATTGACTTGCAAGAGCTGGGCAAGTACGAGC
AGTACATTAAGTGGCCTTGGTACATTTGGCTGGGCTTCATCGCCGGCCTGATCGCCATTGTGATGGTGACCATCATGCT
GTGCTGCATGACATCTTGCTGCTCTTGCCTGAAGGGCTGCTGCTCTTGCGGCTCTTGCTGCAAGTTCGACGAGGACGAC
TCTGAGCCCGTGCTGAAGGGCGTGAAGCTCCACTACACACGGGATCC
